# A 10-year bibliometric analysis in the field of osteosarcoma treatment from 2014 to 2023

**DOI:** 10.1007/s12672-025-02007-2

**Published:** 2025-02-28

**Authors:** Yiguo Shen, Xiaobo Shao, Jiansong Chen, Xin Tang

**Affiliations:** Department of Orthopedics, Children’s Hospital, Zhejiang University School of Medicine, National Clinical Research Center for Child Health, Hangzhou, 310052 China

**Keywords:** Bibliometrics, Osteosarcoma, CiteSpace, Web of Science

## Abstract

**Objective:**

This paper aims to explore the research hot spot and development trend in osteosarcoma treatment using a bibliometric method based upon Web of Science Core Collection (WoSCC) platform over the last decade.

**Methods:**

The literature related to osteosarcoma and cure which were published from January 2014 to December 2023 were retrieved from the database of WoSCC and made an overall analysis for the papers published including number of articles, distribution of countries and institutions, author information, and keywords, with the CiteSpace 6.2.R5.

**Results:**

A total of 3131 papers were retrieved, including 2601 articles and 530 reviews, and the number of papers published has been increasing year by year in the last decade. There were 415 countries and 10,719 research institutions participating into the study. China’s output of literature was the highest relying on its 1490 papers published, followed by The United States (548 papers). Shanghai Jiaotong university had the largest number of papers published (121 papers) and Central South University ranked second (82 papers). A total of 16,816 authors participated in the study. The number of the paper published by Massimo Serra of the Rizzoli Orthopaedics Institute was the largest (27 papers), followed by Dominique Heymann of the University of Sheffield (20 papers). The visualization analysis of keywords by CiteSpace software showed that the drug resistance, drug delivery, tumor tissue engineering and gene expression have become hotspots in the field of osteosarcoma treatment. Drug resistance significantly limits the effectiveness of current cancer treatments. Drug delivery technology not only enhances the targeting and efficacy of drugs but also helps to overcome drug resistance. The stem cells, targeted therapy, and tumor microenvironment represent the new research trends. In particular, the tumor microenvironment plays a key role in tumor development, progression, and drug resistance, and it offers numerous potential therapeutic targets.

**Conclusion:**

Our investigation has identified key research foci and hotspots in osteosarcoma treatment, including drug resistance mechanisms, innovations in drug delivery technology, stem cell development, tumor microenvironment analysis, the development of novel therapies, and the clinical translation of tumor tissue engineering.

**Supplementary Information:**

The online version contains supplementary material available at 10.1007/s12672-025-02007-2.

## Introduction

Osteosarcomas (OS), as the most common primary mesenchymal tissue-derived highly-malignant bone tumor in children and adolescents, accounts for 35% of primary bone malignancies, with an annual global incidence of 3.4 per 1 million people [1]. OS is characterized by rapid growth, strong invasiveness, high malignancy, susceptibility to lung metastasis, high chemotherapy resistance and low survival rate [2]. The current standard of care (neoadjuvant chemotherapy + surgery + postoperative adjuvant chemotherapy) has achieved a 5-year survival rate of 60–70% for patients [3]. Despite continuous optimization of OS treatment over the past decade, there has been no substantial breakthrough in outcomes or survival rates. Notably, the 5-year survival rate for patients with metastatic disease at initial diagnosis or those who relapse after postoperative chemotherapy remains at a mere 20% [4]. In recent years, emerging therapies such as targeted and immunotherapy have garnered significant attention in osteosarcoma research. Numerous clinical trials related to these therapies have been approved and are progressing steadily. Meanwhile, various novel drug delivery systems, including nano-liposomes and 3D-printed drug delivery scaffolds, have been developed. These systems enhance drug efficacy, reduce drug resistance, and minimize side effects, showing great potential for overcoming the current treatment challenges in osteosarcoma. However, the management of OS continues to face considerable obstacles. With ongoing research in this field, a large number of papers have been published in the past 10 years. Therefore, a comprehensive review of these publications is essential to gain a thorough understanding of the research developments in this area.

CiteSpace software helps create networks of authors, countries, institutions, and keywords in research papers. This allows for a quick and clear understanding of key knowledge in specific areas and helps identify new research directions and trends [5]. The WoSCC database, which encompasses 12,000 influential, high-quality journals from around the globe, is considered one of the most authoritative and optimal databases for bibliometric analysis of scientific publications. Consequently, based on the WoSCC database, this paper employed the CiteSpace software to make a scientometrics analysis of the papers which are related to OS treatment and published in the past 10 years. By presenting the current state and hotspots in this field through knowledge graphs, the study aims to uncover future research trends, thereby providing valuable references for scientific research and applications.

## Materials and methods

### Data sources and collection

**Fig. 1:**
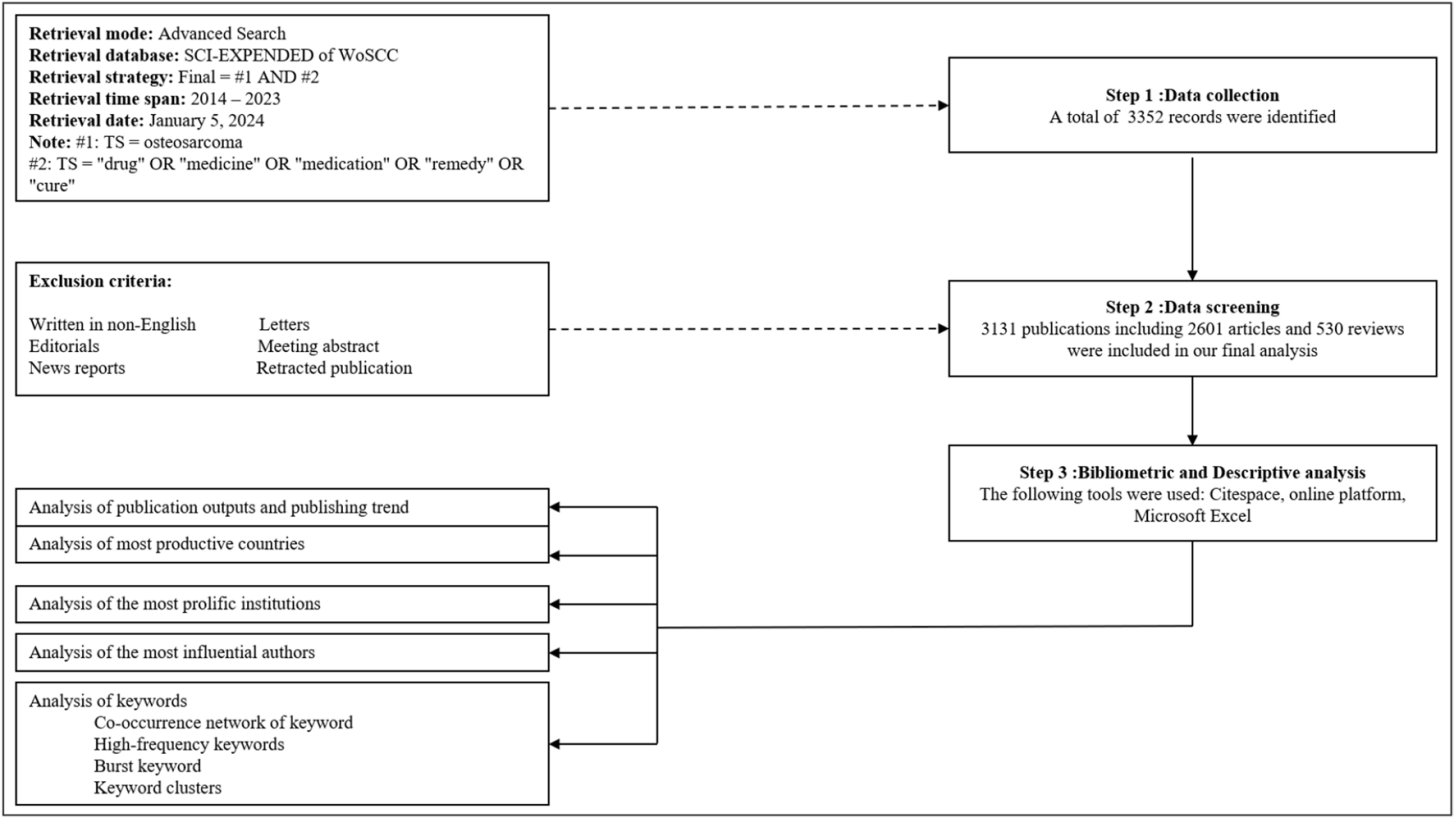
Flowchart of literature selection and data analysis

Based on the WoSCC database, the retrieval strategy was as follows: (TS = osteosarcoma) AND (TS = ’’drug’’ OR ‘‘medicine’’ OR ‘‘medication’’ OR ‘‘remedy’’ OR ‘‘cure’’), with paper types limited to ‘‘Article’’ and ‘‘Review’’. The language was restricted to ‘‘English’’. The search spanned from January 2014 to December 2023 and was conducted on January 5, 2024. This search was limited to original articles and systematic reviews, excluding publication types such as letters, editorials, meeting abstract, and news reports. The final dataset was initially imported into CiteSpace (version 6.2.R5) software to eliminate duplicates. Subsequently, two researchers (Shen and Shao) independently reviewed and screened the original data, with a third researcher (Chen) resolving any discrepancies. (Fig. 1).

### Analysis method

The papers retrieved from the WoSCC database were converted to txt format, and "all recorded and cited references” were exported. Excel 2020 was used to create a list describing the general conditions of the papers. CiteSpace (version 6.2.R5) was employed to analyze the countries, regions, institutions, authors and keywords in the papers and to generate a knowledge map. The software was configured with a time span of 2014–2023, using one time slice per year, and set the threshold to Top 100. When the analysis content network nodes were too dense and unfocused, the Pruning‒Pathfinder algorithm was selected for pruning. Clustering algorithms based on keywords and log-likelihood ratios (LLR) were utilized for cluster analysis. For cluster analysis, Q value (modularity value) > 0.3 indicates significant cluster structure, S value (silhouette value) > 0.5 indicates reasonable clustering, and S value > 0.7 indicates that the clustering is efficient and convincing. The Q value indicates the degree to which nodes form tightly connected groups in a network. The S value measures a node's fit within its cluster and its mismatch with other clusters.

## Results

### Publishing trend

**Fig. 2:**
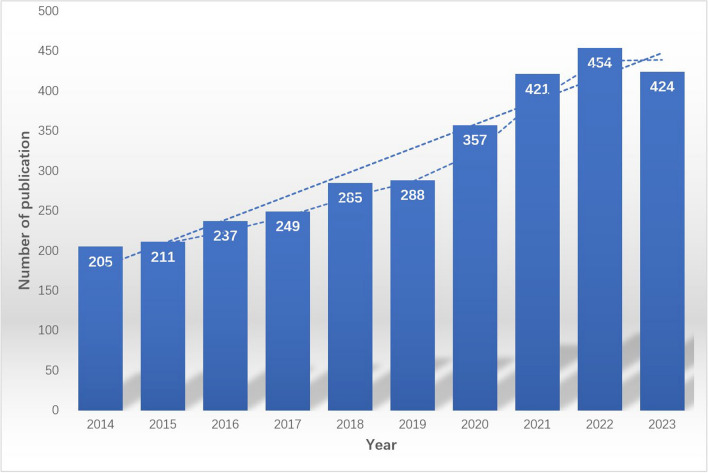
Number of publications

Based on the aforementioned retrieval strategy, a total of 3352 papers were retrieved from the WoSCC database. After applying the exclusion criteria, screening, and removing duplicates, 3,131 papers relevant to osteosarcoma (OS) treatment were ultimately selected for analysis, comprising 2,601 articles and 530 reviews. Over the past decade, there has been a consistent annual increase in the number of publications related to OS treatment, indicating a stable growth trend. Specifically, the total number of papers published in 2022 (454) exceeded that of 2014 (205) by more than 2.2 times, highlighting significant development potential in this research area. The drop in publications in 2023 might be due to when we collected the data—January 5, 2024. Typically, there is a lag of several weeks to months between article acceptance by journals and indexing in WoSCC. Thus, data retrieved at a fixed point in time through WoSCC will inevitably miss some recently published articles. Moving forward, we will seek more optimal data collection timings and incorporate additional databases, such as PubMed. (Fig. 2).

### Distribution of research countries and institutions

**Fig. 3:**
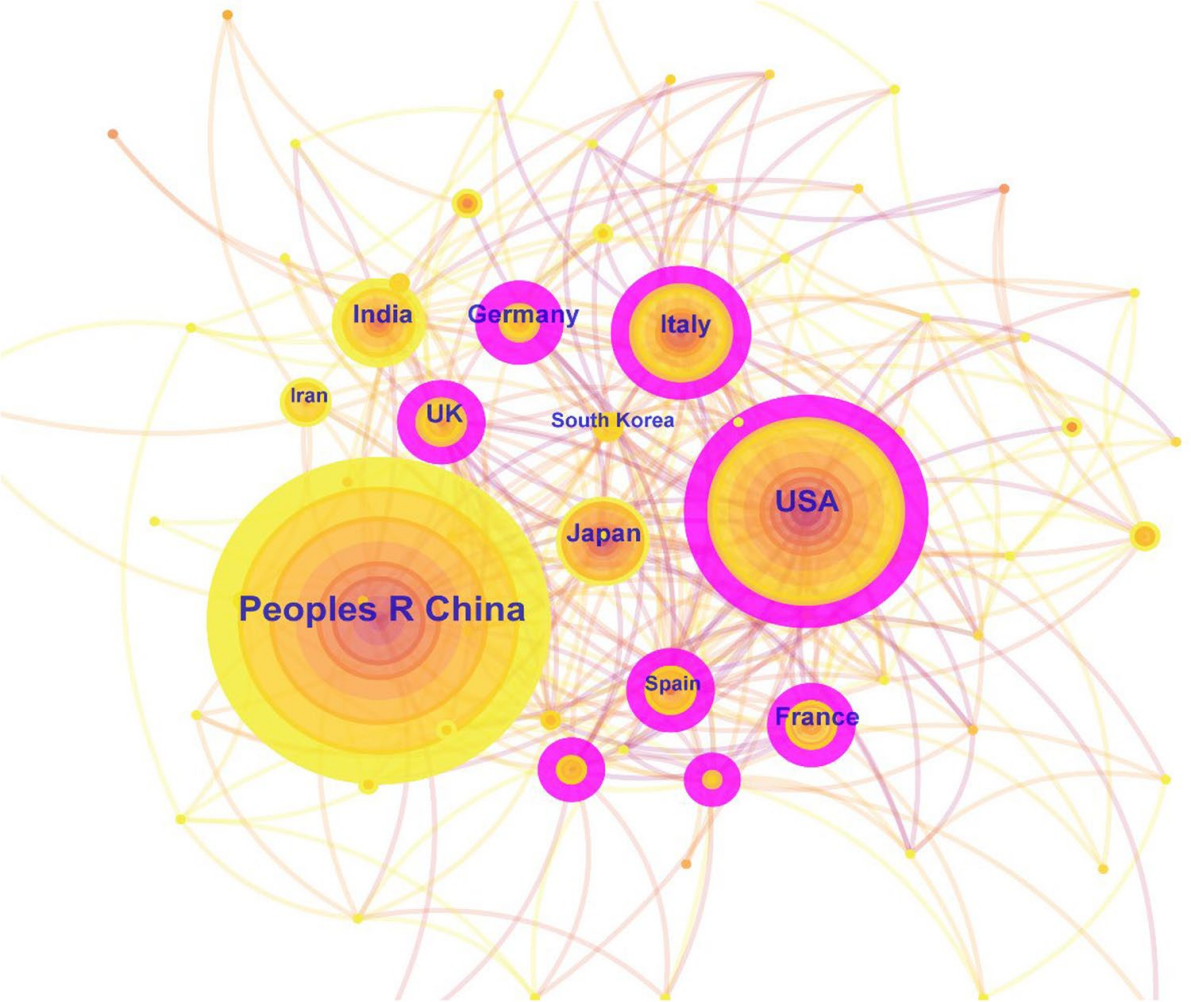
The network map of co-country in OS treatment

A total of 415 countries participated in the research on OS treatment from 2014 to 2023. We used the CiteSpace software to obtain a visualization co-occurrence map of the counties (Fig. 3). The top 5 countries in terms of the number of published papers were as follows: China (1490 papers), the United States (548 papers), Italy (208 papers), India (145 papers), and Japan (144 papers). The top 5 countries with the highest centrality were as follows: USA (0.24), Germany (0.17), UK (0.16), Italy (0.15) and Canada (0.15). The United States and Italy were at the hub of this field owing to their leading number of published papers in the world and close cooperation with other countries. Although China has the largest number of published papers, it has relatively less cooperation with foreign countries.

**Fig. 4:**
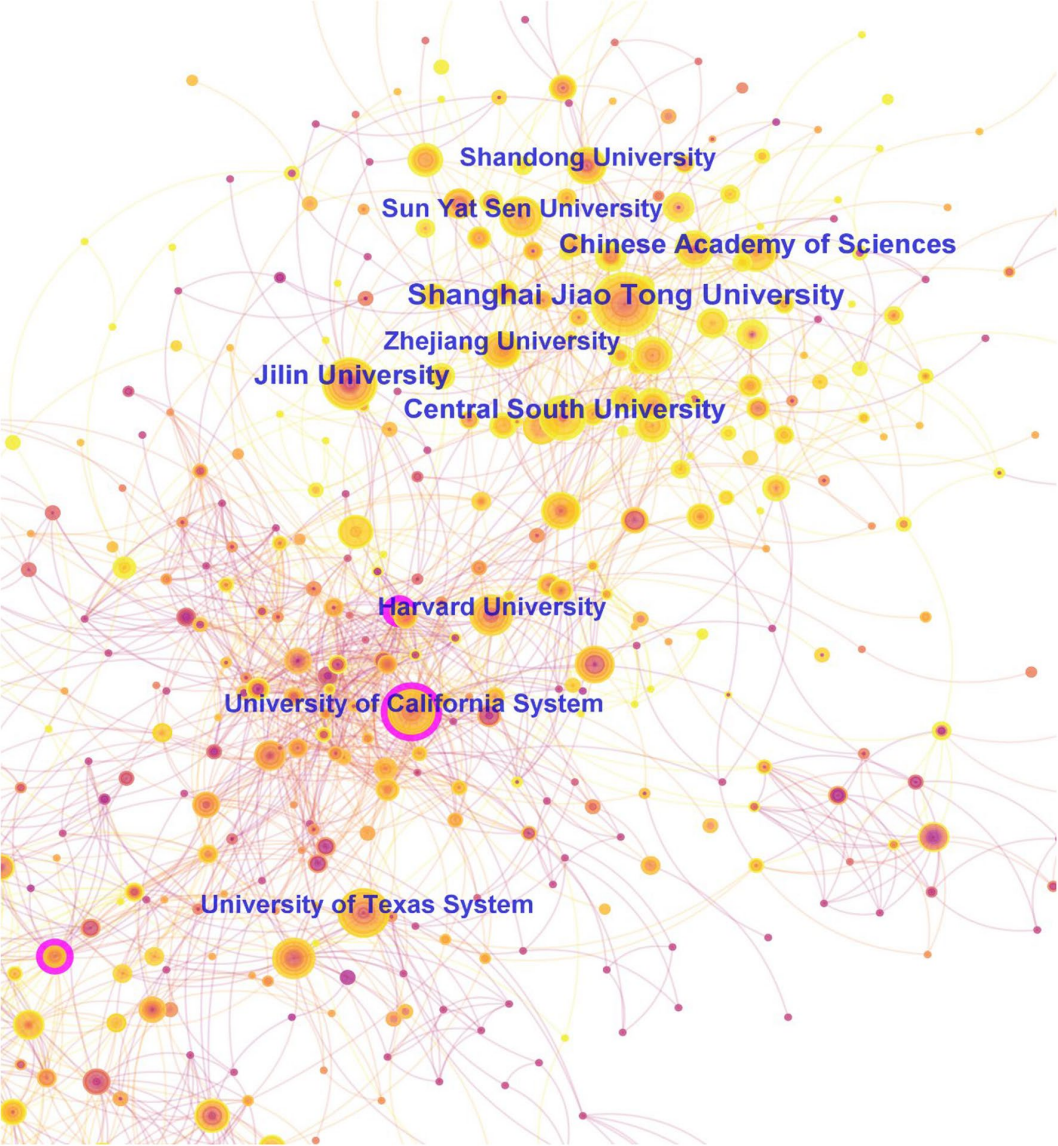
The network map of co-institutions in OS treatment

The number of published papers is an important index to measure the strength of scientific researches. Over the past decade, a total of 10,719 institutions around the world had been involved in studies on OS treatment. Using CiteSpace software, we generated a visual co-occurrence map of these institutions (Fig. 4). The top 5 institutions in terms of the number of published papers were Shanghai Jiao Tong University (121 papers), Central South University (82 papers), Jilin University (76 papers), Chinese Academy of Sciences (65 papers), and University of Texas (63 papers). The top-4 research institutions were all from China, indicating that China had made significant progress in OS treatment over the past 10 years. The institutions with centrality > 0.1 were only University of London (0.19), University of California (0.18), Egyptian Knowledge Base (0.12) and Dana-Farber Cancer Institute (0.1), suggesting that there was limited collaboration among global research institutions in the field of OS treatment.

### Analysis of research authors

In 2014 to 2024, 16,816 authors around the world contributed to the papers on the OS treatment. The top 5 authors in terms of the number of published papers were Massimo Serra (27 papers), Dominique Heymann (20 papers), Zhang Tao (19 papers), Bahman Yousefi (18 papers), and Hua. Yingqi (18 papers). Massimo Serra has made significant progress in the immunotherapy, molecular mechanisms, and drug delivery systems for osteosarcoma. He discovered that chemotherapy-resistant osteosarcoma is highly sensitive to IL-15-activated allogeneic and autologous NK cells, supporting the therapeutic potential of NK cells or NK cell activators in high-grade osteosarcoma patients. He also investigated the intrinsic and extrinsic effects of the RANKL/RANK signaling pathway in osteosarcoma, from tumor initiation to lung metastasis. Additionally, he contributed to the development of various drug delivery systems, such as doxorubicin-loaded calcium phosphate bone cement and sequentially responsive injectable hydrogels carrying NOX4 inhibitors and doxorubicin liposomes. According to Price's law, authors with 11 or more published papers are considered core authors. Among the 16,816 authors, 27 core authors published 420 papers, which accounted for 13.41% of the total number of published papers.

**Fig. 5:**
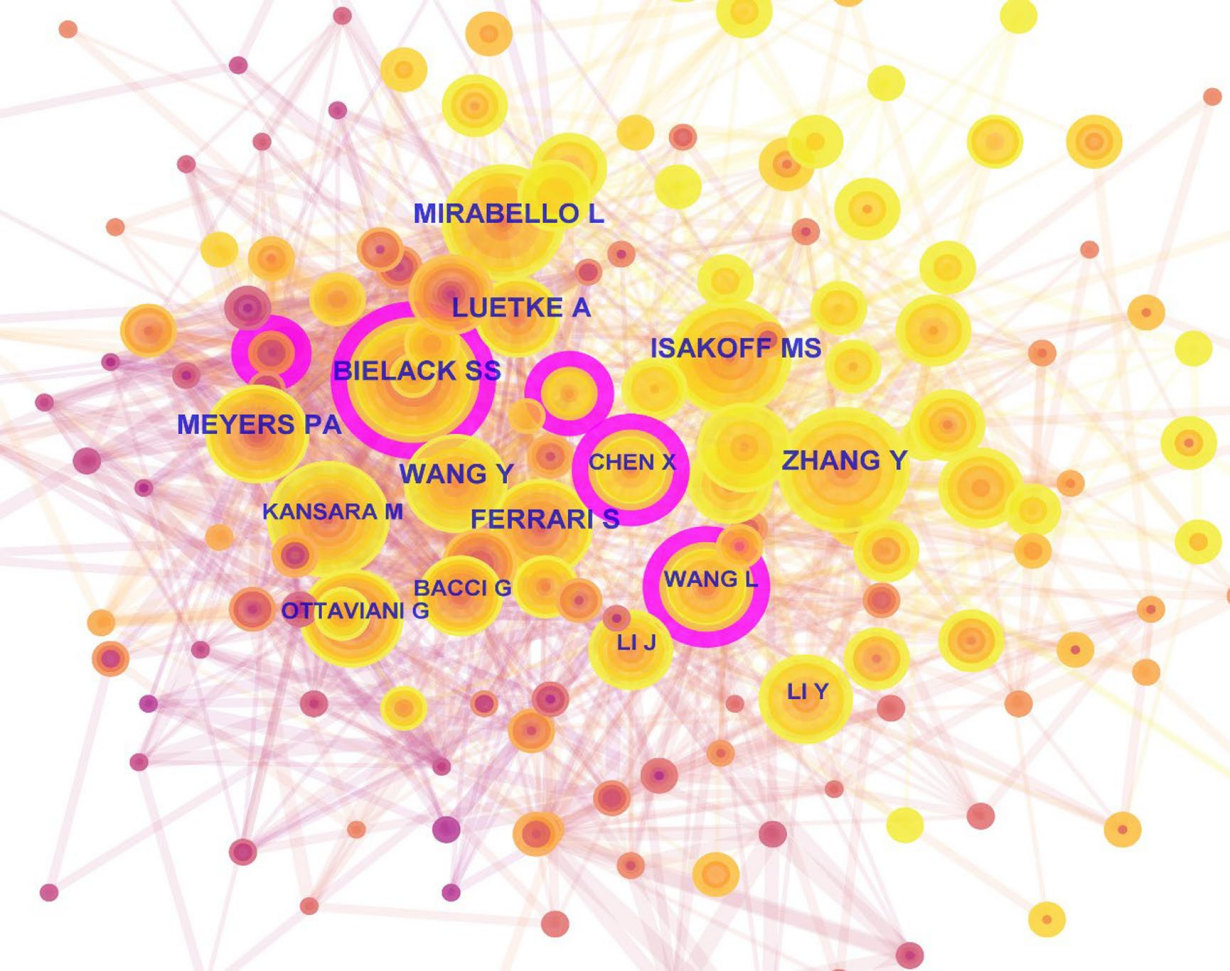
The network map of co-cited authors in OS treatment

CiteSpace was utilized for author co-citation analysis to get a visualization map of author co-citation (Fig. 5). The authors with top 5 citation frequency were MIRABELLO L (342 times), ZHANG Y (336 times), BIELACK SS (303 times), ISAKOFF MS (267 times) and WANG Y (263 times). Lisa Mirabello's research primarily focuses on the genetic susceptibility of childhood cancers and the genomics of osteosarcoma. She led the first international multi-institutional GWAS on osteosarcoma risk, identifying genetic variants associated with osteosarcoma susceptibility. She also spearheaded the first GWAS on osteosarcoma metastasis, uncovering novel genetic loci linked to metastatic disease. These findings have provided valuable genomic data and molecular insights into osteosarcoma, offering new grounds for its prevention and early diagnosis. A purple circles was displayed outside the node for author's BIELACK SS, indicating a centrality greater than 0.1. This suggests that the papers published by this author frequently serve as a bridge to connect other papers and possess strong cohesion and significance in the field of osteosarcoma (OS) treatment.

### Analysis of keywords

**Fig. 6:**
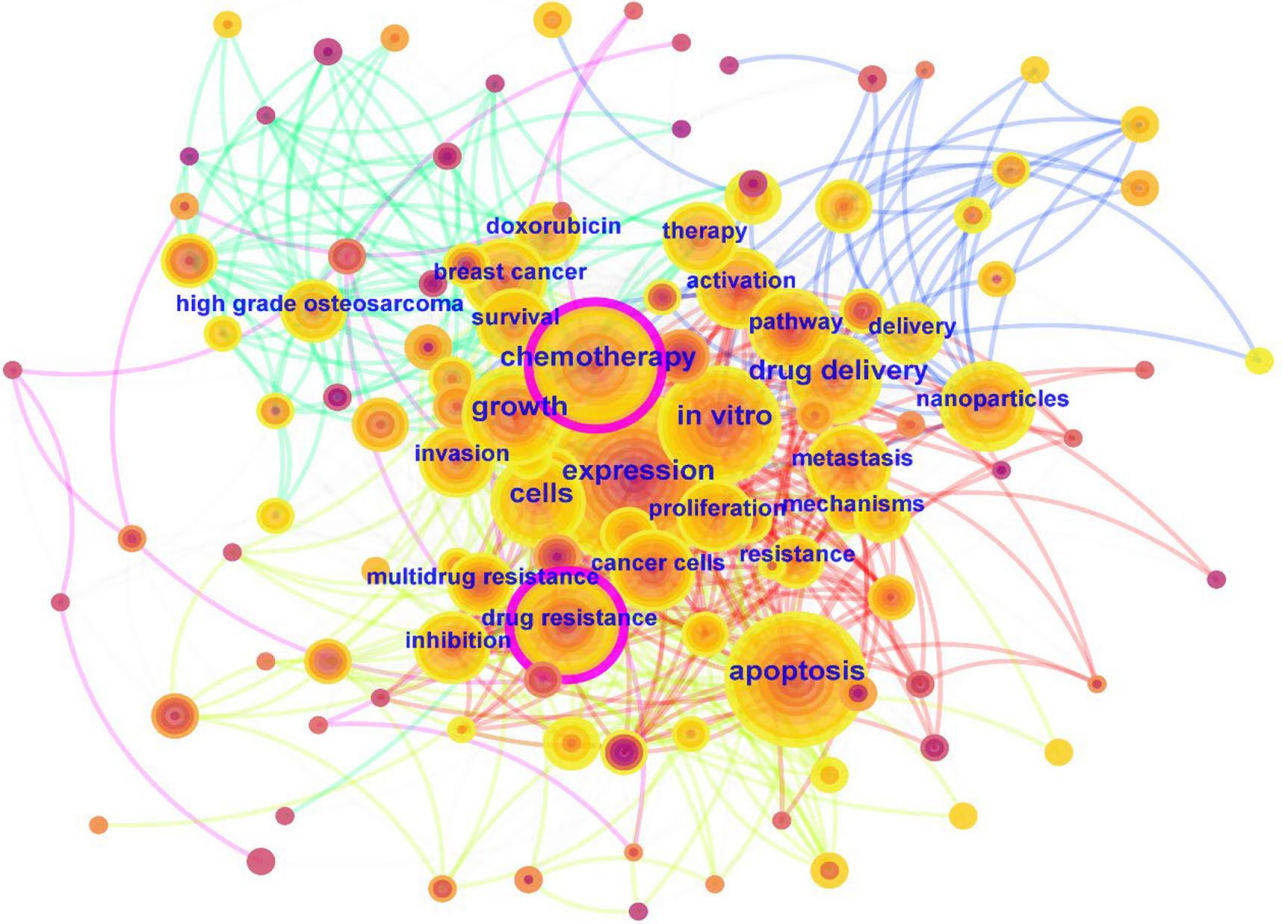
. High-frequency keywords co-occurrence map in OS treatment

Keywords are typically used to succinctly summarize the content of papers, making them well-suited for identifying research hotspots and trends. The CiteSpace software was employed for keyword analysis to generate a co-occurrence map of keywords in the field of OS treatment (Fig. 6). We consolidated keywords with similar meanings; for instance, we merged ‘‘drug resistance’’ and ‘‘resistance’’ into a single term, ‘‘drug resistance,’’ and combined ‘‘inhibition" and ‘‘inhibitor’’ into ‘‘inhibition’’. Additionally, we excluded search terms and general keywords as such as ‘‘cancer’’, ‘‘osteogenic sarcoma’’, ‘‘human OS’’ and ‘‘bone cancer’’. Finally, the top 20 keywords in the field of OS treatment were obtained, as shown in Table 1. Based on the ranking of these keywords, drug resistance and drug delivery represent the important directions in the field of OS treatment.

**Table 1:** Top 20 high-frequency keywords for OS treatment

Ranking	Frequency	Keywords
1	487	Expression
2	449	Drug resistance
3	399	Drug delivery
4	379	Apoptosis
5	349	In vitro
6	324	Chemotherapy
7	256	Cells
8	252	Growth
9	222	Proliferation
10	203	Cancer cells
11	198	Breast cancer
12	190	Metastasis
13	187	Nanoparticles
14	183	Activation
15	182	Doxorubicin
16	170	Survival
17	159	Inhibition
18	144	Pathway
19	118	Multidrug resistance
20	115	Invasion

**Fig. 7:**
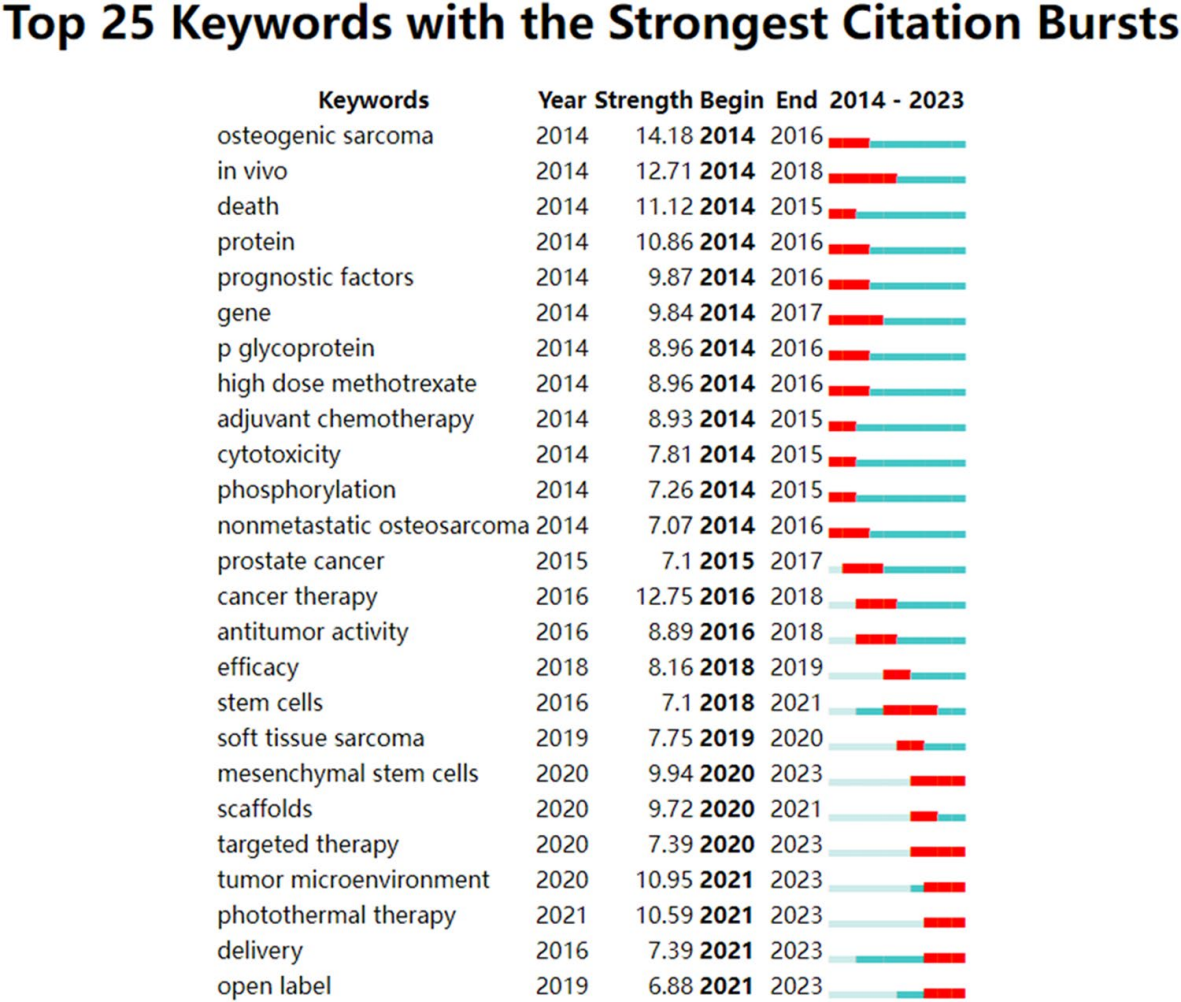
The top 25 keywords with the strongest citation bursts

A burst keyword is one whose frequency of occurrence increases significantly within a specific period, indicating the cutting edge of research during that time and reflecting emerging research hotspots and trends. The 25 keywords with the strongest citation bursts in the field of OS treatment are shown in Fig. 7. The early studies in this field focused on the adjuvant chemotherapy, mechanism of action, prognostic factors, mortality and genes. After 2017, ‘‘stem cells’’ and ‘‘anti-tumor activity’’ became the hot spots; In the past 3 years, the study focus was mainly on mesenchymal stem cells, scaffolds, targeted therapy, tumor microenvironment, photothermal therapy, and drug delivery. And the keyword “stem cells” (including mesenchymal stem cells) has spurted for a longest period since 2018.

**Fig. 8:**
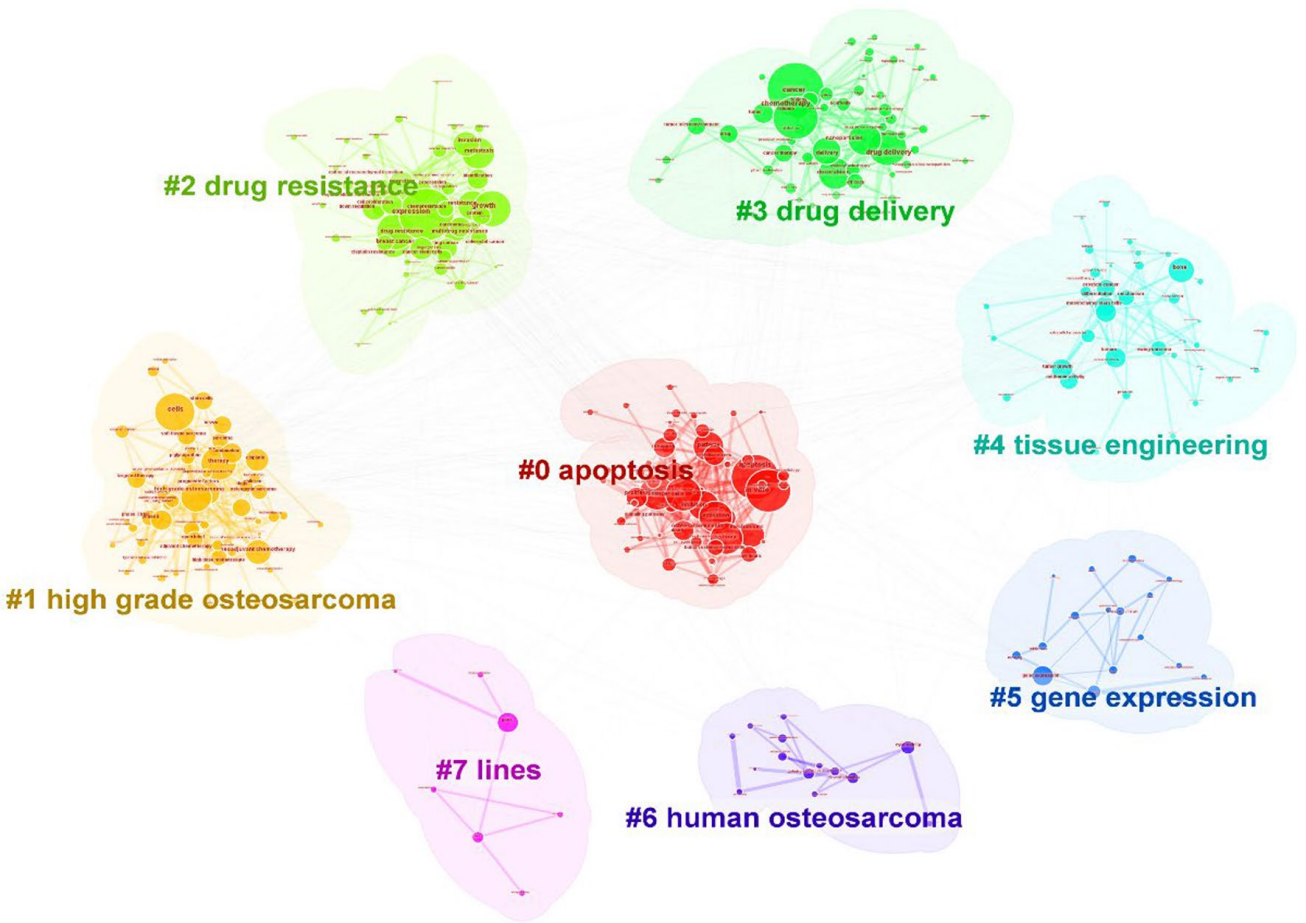
The cluster network map of keyword in OS treatment

We conducted a cluster analysis on the keyword co-occurrence network, resulting in a keyword cluster map with a Q value = 0.4122 (> 0.3) and an S value = 0.7236 (> 0.7) (Fig. 8). The 8 keyword clusters in the map are Cluster #0 cell apoptosis, Cluster #1 High-grade osteosarcoma, Cluster #2 drug resistance, Cluster #3 Drug delivery, Cluster #4 Tissue engineering, Cluster #5 gene expression, Cluster #6 Human osteosarcoma, and Cluster #7 lines. The smaller the cluster number, the more keywords it contains. These keyword clusters reflect the focuses in the field of OS treatment in the recent 10 years.

**Fig. 9:**
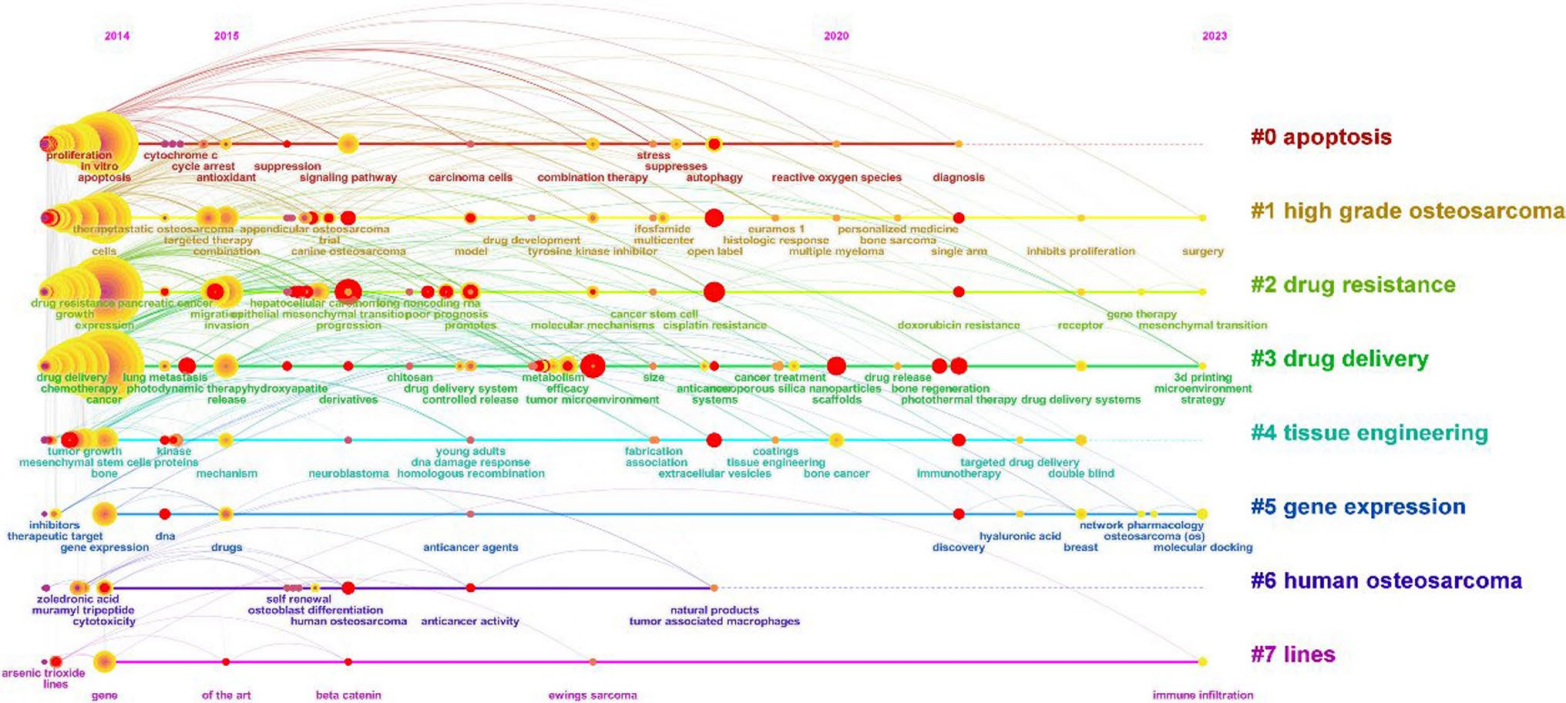
The timeline map of keyword clusters in OS treatment

Using the publication year of the cited papers as the X-axis and the cluster number as the Y-axis, we obtained a time series for the field of osteosarcoma (OS) treatment (Fig. 9). Clusters such as #1 high-grade osteosarcoma, #2 drug resistance, and #3 drug delivery have many literatures and the widest span, and are still in evolution. #1 high-grade osteosarcoma: from far to near including non-metastatic, targeted therapy, combination, model, drug development, multicenter, histological response, personalized medicine, inhibits proliferation, surgery and other keywords; #2 Drug resistance: from far to near including drug resistance, migration, invasion, epithelial-mesenchymal transformation, non-coding RNA, molecular mechanism, cancer stem cell, cisplatin resistance, doxorubicin resistance, gene therapy, mesenchymal transformation and other keywords. #3 Drug delivery: from far to near including chemotherapy, photodynamic, release, hydroxyapatite, chitosan, drug delivery system, tumor microenvironment, silica nanoparticles, scaffold, bone regeneration, photothermal therapy, and 3D printing. These keywords represent the clusters in which they are located are in continuous evolution, and many of them also appear as burst keywords, indicating the research direction and hotspots during different periods.

## Discussion

According to the latest global cancer burden indicators 2020 released by the International Agency for Research on Cancer (IARC), China has become a veritable ‘‘cancer country’’, ranking first in the world in both new cases and deaths. OS is one of the leading causes of disability and death among children and adolescents, imposing a heavy economic burden on society and families. The exploration of more effective treatments is not only an urgent clinic challenge, but also an important topic in medical study. Therefore, it is of great significance to comprehensively review and investigate the literature in the field of OS treatment.

## Distribution of research strength

Through visualization research, this study demonstrates that the literature on the OS treatment had shown a steady upward trend over the past decade, indicating the scholars have paid considerable attention to this field. China had the largest number of papers published related to the OS treatment, more than twice as many as the United States, which ranked second. This may be due to China's large population and its research evaluation system, which focuses on the number of individual publications and impact factors. Shanghai Jiao Tong University, Central South University, Jilin University and the Chinese Academy of Sciences were the top 4 research institutions in terms of number of the paper published. Obviously, China has put a lot of effort in the field of OS treatment and achieved fruitful achievements, being a leader in the world. The co-occurrence network of authors indicates that numerous collaborative groups have formed globally in the field of osteosarcoma (OS) treatment. Compared to their European and American counterparts, Chinese researchers have relatively limited international collaboration, due to language and cultural differences, constraints in research resources and platforms, geopolitical tensions, and the decline in international academic exchanges following the COVID-19 pandemic. Massimo Serra of Rizzoli Institute of Orthopedic Research published the most papers. Lisa Mirabello of the National Cancer Institute of the United States was the most frequently cited author in the field. Stefan Bielack of Stuttgart Cancer Center was among the top in the world for both citation frequency and intermediary centricity. The papers published by these three authors represented the latest developments in the field to some extent.

## Hot spots and cutting-edge trends in research

Keywords are typically used to succinctly summarize the content of papers, and the high-frequency keywords can reflect the study status and hot topics in the field to a certain extent and the burst keywords can effectively grasp the frontier in a certain period [5]. In this study, by integrating high-frequency keywords and keyword clusters, we identified drug resistance, drug delivery and tissue engineering as the three major research directions in OS treatment. The focus of gene expression and tumor research is on assessment indicators such as cell apoptosis, survival, and metastasis. The time graph showed that the most of clusters are still in evolution, in particular, the drug resistance and drug delivery have been the study focuses. As evident from the top 25 bursting keywords, terms such as stem cell, scaffold, targeted therapy, tumor microenvironment, photothermal therapy, and drug delivery are increasingly mentioned and continue to grow rapidly, representing cutting-edge research trends.

## Treatment status and drug resistance of OS

### Chemotherapy

“Neoadjuvant chemotherapy + surgery + postoperative adjuvant chemotherapy” remains the primary treatment option for OS. Neoadjuvant chemotherapy significantly rises relapse-free survival (RFS) of patients with nonmetastatic disease and attains a minimum of 90% necrosis on the surgically resected tumor [6]. High-dose methotrexate (HD-MTX), doxorubicin (ADM), cisplatin (DDP), and IFO (IFO) are recognized as stable and effective first-line chemotherapy drugs [7]. Clinically, these four drugs are combined in various doses, combinations, and sequences to develop different chemotherapy regimens aimed at improve limb preservation rate and 5-year survival rate, as well as reducing drug resistance. Currently, several novel chemotherapy drugs are under investigation, with gemcitabine (GEM), Docetaxel (TXT), and their combination receiving the most attention [8]. Numerous clinical studies have reported that the combination of TXT and GEM can effectively treat OS (recurrent/refractory and progressive), which is the most potential new treatment strategy [9–11]. A single-arm Phase II study conducted by Martin-Broto RA, Valverde C, et al., showed that gemcitabine plus sirolimus in combination achieved significant results in the treatment of metastatic/recurrent OS and may be a safer alternative to gemcitabine and docetaxel [12]. In addition, efforts to improve the sensitivity of tumor cells to chemotherapeutic drugs through the regulation of related genes, as well as to enhance the cytotoxicity of these drugs, have been widely pursued. Park et al. found that the sensitivity of OS cells to doxorubicin could be significantly improved by the application of Olaparib (a PARP1 inhibitor of DNA-damage repair related genes) [13]. Pan et al. revealed that the transcription factor Sp1 increases the pyroptotic level and the sensitivity of cells to chemotherapeutic drugs under stimulated conditions by promoting the transcription of GSDME [14]. Huang et al. demonstrated that PTEN protein in myeloid cells can bolster chemotherapy-induced anti-tumor immunity by facilitating the NLRP3 inflammasome within the immune microenvironment [15].

### Drug resistance

Drug resistance has consistently been a major problem facing cancer treatment, almost all drugs as patients use time, cancer cells will develop resistance to drugs, making the drug lose its effect, and eventually lead to cancer recurrence, rapid disease progression, and even death [16]. Tumor drug resistance arises from the interaction of multiple pathways and factors, making its mechanism highly complex. Previous studies have found that drug resistance in OS is associated with gene mutation, drug efflux, cell detoxification, DNA damage repair, apoptosis, autophagy, and tumor microenvironment changes [17]. Exosomal microRNAs play a significant role in the occurrence and progression of OS. Studies have proved that the killing effect of chemotherapy drugs on OS cells can be enhanced by up-regulating the expression of miR-124 [18], miR-15 [19], or miR-34a [20]. Some studies have indicated that alterations in the mitochondrial network, membrane potential, and quality are associated with tumor chemotherapy resistance or cancer aggressiveness. Analysis of mitochondrial profile types can identify the altered pathways, which is helpful to screen the target of drug-resistant cancer cells and promote the development of new drugs [21, 22]. Natural compounds have also shown potential ability to overcome the resistance problem. Studies have demonstrated that the combination of drug therapy with evodiamine can overcome chemotherapy and radiation resistance exhibited by cancer cells, thereby enhancing the anticancer efficacy of chemotherapeutic agents across various types of cancer.[23]. Curcumin is considered a promising anticancer drug candidate, exhibiting significant anticancer effects by targeting various cell signaling pathways [24]. Other researchers have used CRISPR-Cas9 gene editing technology to specifically repair gene mutations in tumor cells, thereby restoring their sensitivity to treatment [25]. Therefore, exploring the potential mechanism of OS drug resistance, overcoming this resistance, and discovering new effective drugs remain key challenges and focal points in OS treatment research, offering vast potential for further investigation.

## Emerging therapy

### Targeted therapy

In recent years, targeted therapy, which inhibits tumour growth at the cellular and molecular levels by affecting known gene loci, has been widely applied in the treatment of OS [16]. Currently, studies on targeted therapy for OS primarily focus on the development of multi-target anti-angiogenesis small molecule drugs [26]. Multiple Tyrosine kinase inhibitors (TKIs) have been demonstrated to have efficacy in patients with relapsed and/or unresectable disease, including sorafenib (4-month progression-free survival (PFS) of 46%) [27], regorafenib (median PFS of 3.6 months versus 1.7 months with placebo) [28], cabozantinib (4-month PFS of 71%) [29, 30], lenvatinib (4-month PFS of 33%) [31] and pazopanib (median PFS of 5.5 months in patients with cancers of mixed histologies, 50% of which were osteosarcomas) [32]. In a single-arm multicenter trial (ALTER-S002), the combination of anlotinib, doxorubicin, and cisplatin was found to be effective for treating stage IIB classic osteosarcoma of the limbs, with manageable side effects [33]. Although the affinity of these compounds for different receptor tyrosine kinases (RTKS) varies, VEGFR is a common target for all of these TKIs, and have generated excitement in the field.

Drug resistance is a significant issue that limits the efficacy of targeted drug therapies. Tumor resistance is related to many factors, including gene mutation and expansion, apoptosis and autophagy disorder, among which the gene mutation is the main cause [34, 35]. Hussain et al. suggested that interfering with the signaling pathways involved in targeted therapies may help reduce drug resistance [36]. Pottier et al. proposed that the combined application of different types of tyrosine kinase receptor inhibitors could not only inhibit drug resistance to a certain extent, but also reduce its toxicity [37].Other researchers have suggested that combining targeted therapies with immunotherapy may help mitigate drug resistance and reduce adverse reactions [38, 39]. In conclusion, TKIs may serve as the next alternative to be combined with pre-chemotherapy, either as maintenance therapy or through the development of new strategies to safely integrate them into current chemotherapy regimens.

### Immunotherapy

The tumor immune microenvironment (TIME) of OS typically exhibits immunosuppressive traits, which pose challenges for immunotherapy. However, modulating the tumor microenvironment, such as reversing the activation state of cancer-associated fibroblasts (CAFs), can increase the infiltration of effector T cells into tumors, thereby enhancing the efficacy of immunotherapy [40]. Researchers have devised a sequentially responsive injectable nanocomposite hydrogel system that reverses the activation of CAFs, improves tumor fibrosis, and further promotes effector T cells infiltration, effectively curbing osteosarcoma growth [40]. Immune checkpoint inhibitors, such as PD-1/PD-L1 inhibitors, have achieved significant therapeutic effects in various solid tumors [41], but their application in OS is still in the exploratory stage. Numerous studies have shown that the combination of immune checkpoint inhibitors with chemotherapy or targeted therapy can markedly improve treatment outcomes [42]. A clinical trial of camrelizumab (a PD-1 inhibitor) in combination with chemotherapy revealed that about 48.4% of patients had a chemotherapy necrosis rate of ≥ 90%, with a 2-year overall survival rate reaching 86.7% [43]. Adoptive cell therapy has achieved certain results in OS. Although the technology for isolating and expanding TILs from OS patients is still immature, studies have shown that the proportion of TILs is positively correlated with patient prognosis [44]. Other researchs have found that CAR-T cell therapy targeting specific antigens, such as anti-HER2 [45], anti-GD2 [46], CAR-NK [47] and CAR-M [48], has shown preliminary efficacy in clinical trials. Emerging immunotherapeutic approaches, including tumor vaccines, targeted cytokines, and bispecific antibodies, are also being actively developed. These strategies enhance the body's immune response, overcome immune tolerance, and prevent immune escape, significantly improving the immune response of patients [44]. The OS microenvironment contains multiple immunosuppressive pathways, and the response rate to monotherapy immunotherapy in OS is low. Combination therapy strategies, such as the combination of immunotherapy with chemotherapy, radiotherapy, or targeted therapy, have shown significant synergistic effects. Lussier et al. demonstrated in an OS mouse model that the combination treatment with anti-PD-L1 and anti-CTLA-4 antibodies completely controlled metastatic OS [49]. In a Phase I/II study (ITCC-050), the combination of lenvatinib and the IE regimen (ifosfamide + etoposide) demonstrated good antitumor activity in patients with recurrent/refractory osteosarcoma, with a median PFS of 8.7 months and a 4-month PFS rate of 51% [50]. We believe that future research directions in osteosarcoma immunotherapy will focus on modulating the tumor microenvironment, combining targeted therapies, CAR-T cell therapy, and developing new immunotherapeutic strategies. Combination therapy based on immunotherapy that converting a immune cold into an inflamed microenvironment may be a future direction.

## Innovative treatment

### Drug delivery technology

Drug delivery technology has garnered widespread attention due to its advantages, such as controlled delivery rate, targeted delivery and prolonged circulation time in vivo. Nanoparticle carriers play a crucial role in drug delivery systems because the enhanced permeability and retention (EPR) effect of tumor cells allows nanoparticles to remain in the tumor microenvironment for extended periods, in addition to their unique charge and targeting properties [51]. Liposomal formulations are the first nanosized drug delivery carriers that have been successfully translated into clinical applications, such as liposomal muramyl tripeptide phosphatidyl ethanolamine (L-MTP-PE) [52], and PEGylated liposomal nanocarrier [53]. Michael et al. developed a switchable bispecific T-cell engager (SiTE) that uses the small molecule drug adamantane (AMD) to control the separation of T cells from tumor cells, effectively preventing off-target toxicity [54]. Polymer has received increased interest in recent years, such as poly lactide-co-glycolic acid (PLGA) and polyethylene glycol (PEG), and hyaluronic acid (HA). They are not only widely used for anti-cancer drug delivery, but are also commonly employed for modifying nanocarriers to enhance their stability, biocompatibility, and specificity [55–57]. Calcium phosphates (CaP) nanoparticles, particularly hydroxyapatite nanoparticles (HANPs), are considered promising bone tissue nanocarriers because they have been shown to preferentially accumulate in bone tissues [58, 59]. CaP-based nanoparticles have been widely used for delivery of anticancer drugs in OS treatment [58–61]. Drug delivery technology has made remarkable breakthroughs in recent years, and several liposomal products based on small molecule drugs have been approved by the US FDA, such as Doxil®, Onivyde®, and Vyxeos®. It is anticipated that their translation into clinical applications will gradually be realized in the near future, thereby benefiting cancer patients.

### Tissue engineering technique

Advances in tissue engineering have led to the development of 3D model structures, such as spheroids and organoids, which more accurately replicate the complex intracellular dynamics and microenvironment of OS [62]. 3D OS cell culture models can enhance the responsiveness of clinical chemotherapy and advance research in personalized cancer medical [63]. The organoid cell 3D model constructed by Ozturk et al. can preserve stem cell phenotypes for a longer duration than traditional two-dimensional (2D) cultures, thereby enhancing the relevance of screening and improving targeting efficiency during drug detection [64]. Compared with traditional 2D and CSC models in vitro, the autophagy level in the 3D bioprinted OS model developed by Lin et al. more closely resembles in vivo autophagy levels, and its drug screening results align with clinical outcomes [65]. He et al. successfully established a patient-derived organoid culture system that simulates pulmonary metastatic OS, which effectively simulates the complex tumor microenvironment while maintaining the histological and molecular properties of the original tumor [66]. However, there is still considerable room for improvement in the biofidelity and robustness of 3D tumor models. Additionally, 3D bioprinted bone tissue engineering scaffolds have introduced new ideas and directions for the treating bone defects and residual tumor cells after OS surgery. Zhang et al. developed a 3D-printed gelatin/shell scaffold, using gelatin with DOX as the core part and SrCuSi4O10 (SC) nanosheets/β-TCP as the shell of the printed filament, which exhibited good osteogenic and antitumor effects [67]. Sarkar et al. encapsulated curcumin in liposomes and then incorporated it into a 3D-printed calcium phosphate scaffold with designed porosity, which both eradicated OS cells and promoted osteoblast proliferation [68]. Many scientists are focusing on developing photothermal composite scaffolds that can promote regeneration and have therapeutic effects in OS treatment [69, 70]. Although 3D bioprinted bone tissue engineering scaffolds have demonstrated significant market potential, no scaffolds have been able to accurately simulate the mechanical and bone conduction properties of natural bone. Furthermore, the mechanisms of interaction between biomaterials and cells require further investigation.

## Limitations

Similar to other scientometric studies, there are several limitations should be addressed in our study. First, our analysis was confined to the WoSCC database, excluding other databases like PubMed and Scopus, which might have led to an incomplete collection of literature. Cross-referencing multiple databases can boost data volume, minimize bias, and offer a more precise analysis of research trends and hotspots, as well as pinpoint interdisciplinary research directions. Second, by focusing solely on articles published in English, we inevitably overlooked research published in other languages. Third, we didn’t exclude articles invited by journals or authored by editorial board members, which could potentially introduce bias. However, given that English is still the lingua franca of global academic research and WoSCC is the most widely used database in scientometric studies, we believe our study still effectively captures the overall landscape and trends in this field.

## Conclusion

Our research indicates that over the past decade, the field of osteosarcoma (OS) treatment has seen significant activity, with evolving research priorities. Initial emphases on chemotherapy, neoadjuvant chemotherapy, mechanisms of action, prognostic factors, and mortality have transitioned towards a focus on drug resistance mechanisms, drug delivery, tissue engineering, targeted therapy, tumor microenvironment, and stem cell development. Given the tumor microenvironment's pivotal role in tumor initiation, progression, treatment response, and resistance, as well as its numerous potential therapeutic targets, we expect it to attract increasing attention in future research.

## Supplementary Information

Additional file1

## Data Availability

Data is provided within the manuscript or supplementary information files.
